# ﻿*Carexquixotiana* (Cyperaceae), a new Iberian endemic from Don Quixote’s land (La Mancha, S Spain)

**DOI:** 10.3897/phytokeys.221.99234

**Published:** 2023-03-14

**Authors:** Carmen Benítez-Benítez, Pedro Jiménez-Mejías, Modesto Luceño, Santiago Martín-Bravo

**Affiliations:** 1 Botany area, Department of Molecular Biology and Biochemical Engineering, Universidad Pablo de Olavide, Seville, Spain Universidad Pablo de Olavide Seville Spain; 2 Botany area, Department of Plant Biology and Ecology, Universidad de Sevilla, Seville, Spain Universidad de Sevilla Seville Spain

**Keywords:** Cytogenetics, Iberian Peninsula, Mediterranean, morphometrics, new species, *Phacocystis*, phylogenetics, *Quercus* forest, taxonomy

## Abstract

Despite centuries of work, the basic taxonomic knowledge of the flora of the Iberian Peninsula is still incomplete, especially for highly diverse and/or difficult genera such as *Carex*. In this study, we conducted an integrative systematic study based on molecular, morphological and cytogenetic data to elucidate the taxonomic status of several problematic *Carex* populations from La Mancha region (S Spain) belonging to Carexsect.Phacocystis. These populations have been traditionally considered of uncertain taxonomic adscription, but close to *C.reuteriana* due to their morphological appearance and ecological preferences. A detailed morphological and cytogenetic study was performed on 16 La Mancha’s problematic populations (Sierra Madrona and Montes de Toledo) to compare them with the other Iberian sect. Phacocystis species. In addition, a phylogenetic analysis was conducted using two nuclear (ITS, ETS) and two plastid (*rpl*32‐*trn*L^UAG^, *ycf*6‐*psb*M) DNA regions, including representatives from all species of sect. Phacocystis. We found a significant degree of molecular and morphological differentiation that supports the recognition of La Mancha’s problematic populations as a new Iberian endemic species, described here as *Carexquixotiana* Ben.Benítez, Martín-Bravo, Luceño & Jim.Mejías. Our results reveal that *C.quixotiana*, unexpectedly, is more closely related to *C.nigra* than to *C.reuteriana* on the basis of phylogenetic relationships and chromosome number. These contrasting patterns reflect the taxonomic complexity in sect. Phacocystis and highlight the need for integrative systematic approaches to disentangle such complicated evolutionary scenarios.

## ﻿Introduction

The Iberian Peninsula is one of the three large peninsulas of Southern Europe projecting into the Mediterranean Sea, and as such, it harbours a high diversity of plant species and endemism relative to the rest of Europe (Myers et al. 2000; Mittermeier et al. 2011; Vargas 2020). Phytogeographically, it is part of the Mediterranean Basin biodiversity hotspot (Myers et al. 2000; Mittermeier et al. 2011). *Carex* L. (Cyperaceae) is among the three most speciose angiosperm genera in the Iberian Peninsula (Aedo et al. 2017). Floras, monographs and checklists published from the second half of the 20^th^ century have progressively increased the number of reported *Carex* native taxa in the Iberian Peninsula (Vicioso 1959: 74 taxa; Chater 1980: 95 taxa; Luceño and Aedo 1994: 98 taxa; Luceño 2008: 101 taxa; Luceño et al. unpublished data: 108 taxa). These studies, along with the finding of new Iberian records (e.g., *C.cespitosa*, Jiménez-Mejías et al. 2007), re-evaluation of neglected taxa (e.g., *C.paui*, Benítez-Benítez et al. 2017; Troia et al. 2018) or even description of new ones (e.g., *C.lucennoiberica*, Maguilla and Escudero 2016; *C.camposii* subsp. tejedensis, Sánchez-Villegas et al. 2022), demonstrate that the taxonomic and biogeographic knowledge of *Carex* in this territory is still in progress.

Section Phacocystis Dumort. (subg. Carex) is among the largest sections of *Carex* (ca. 112 spp. in Roalson et al. 2021), although the number of taxa differs considerably among treatments (see Benítez-Benítez et al. 2021). It has a sub-cosmopolitan distribution that somewhat mirrors that of the whole genus, with higher species diversity in temperate and cold areas in the Northern Hemisphere, but also with several species present in the Southern Hemisphere (Benítez-Benítez et al. 2021). The typical ecological requirements of the section include habitats with high water availability, such as wetlands, river shores, mountain bogs and wet coastal sands, which are mostly in freshwater systems, although a few high latitude species also grow in halophytic environments. In the Iberian Peninsula, it is represented by six species and eight taxa: *C.acuta* L., *C.cespitosa* L., C.elataAll.subsp.elata, C.nigra(L.)Reichardsubsp.nigra, C.nigrasubsp.intricata (Tineo ex Guss.) Rivas Mart., C.reuterianaBoiss.subsp.reuteriana, C.reuterianasubsp.mauritanica (Boiss. & Reut.) Jim.Mejías & Luceño, and *C.trinervis* Degl. Section Phacocystis is one of the most controversial *Carex* groups from a taxonomic perspective, as species in the group are affected by the complex interplay of high intraspecific morphological variability, faint species boundaries, and interspecific hybridization (see references in Jiménez-Mejías et al. 2014a; Benítez-Benítez et al. 2021). In addition, the use of certain species as taxonomic hotchpotches (e.g., *C.acuta*, *C.cespitosa*), and frequent misidentifications during the taxonomic history of sect. Phacocystis, have greatly obscured the knowledge of the group in certain areas (e.g., Jiménez-Mejías and Martinetto 2013; Jiménez-Mejías et al. 2014b). An example of this among the Iberian taxa of sect. Phacocystis may be observed in *C.elata* and *C.reuteriana*, for which different taxa have been miscited or even confused with other species such as *C.acuta* or *C.nigra* (Luceño and Aedo 1994; Jiménez-Mejías et al. 2011, 2020).

**Figure 1.**
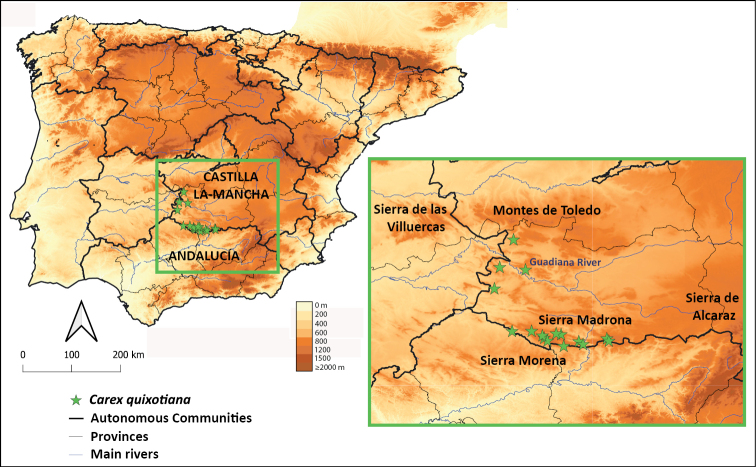
Distribution map representing the studied populations of *Carexquixotiana*.

La Mancha (Fig. 1) is a natural and historical region placed in the south-southeastern limit of the Iberian Central Plateau, mostly belonging to the Guadiana River basin. It is popularly known as the main setting for Miguel de Cervantes’s (1547–1616) novel Don Quixote (de Cervantes 1605, 1615). At an approximate average elevation of 500–600 m, La Mancha has a relatively cold, semi-arid climate and low topographic relief. The region is delimited by Sierra Morena to the south, Montes de Toledo to the north, and Sierra de Alcaraz to the east (Fig. 1), all delimiting the Guadiana basin, and Las Villuercas to the west, across which the River Guadiana stretches towards the Atlantic Ocean. While the central part of La Mancha is mostly formed by carbonate sediments, Sierra Morena and Montes de Toledo are composed of metamorphic siliceous materials, including quartzite, schist and slate. The majority of the present La Mancha landscape is dedicated to agriculture, but potential vegetation would be primarily evergreen sclerophyllous *Quercusilex* forest on the lower lands, and *Q.pyrenaica* and/or *Q.faginea* woodlands on the more elevated ones (Martín Herrero et al. 2003). According to Ramos-Gutiérrez et al. (2021), there are only two plant species endemic to La Mancha, both belonging to the agamospermic genus *Limonium* (*L.pinillense* Roselló & Peris and *L.squarrosum* Erben) and inhabiting inland saltmarshes. There are about 20 species of *Carex* present in La Mancha (Luceño et al. unpublished data). Most of them are limited to habitats with year-round water availability, such as springs, streams or *bonales* (relictic aquifer-fed peat bogs) (Martín-Blanco and Carrasco de Salazar 2005), although a few species also grow on relatively dry soils as part of the Mediterranean forest understory.

**Figure 2.**
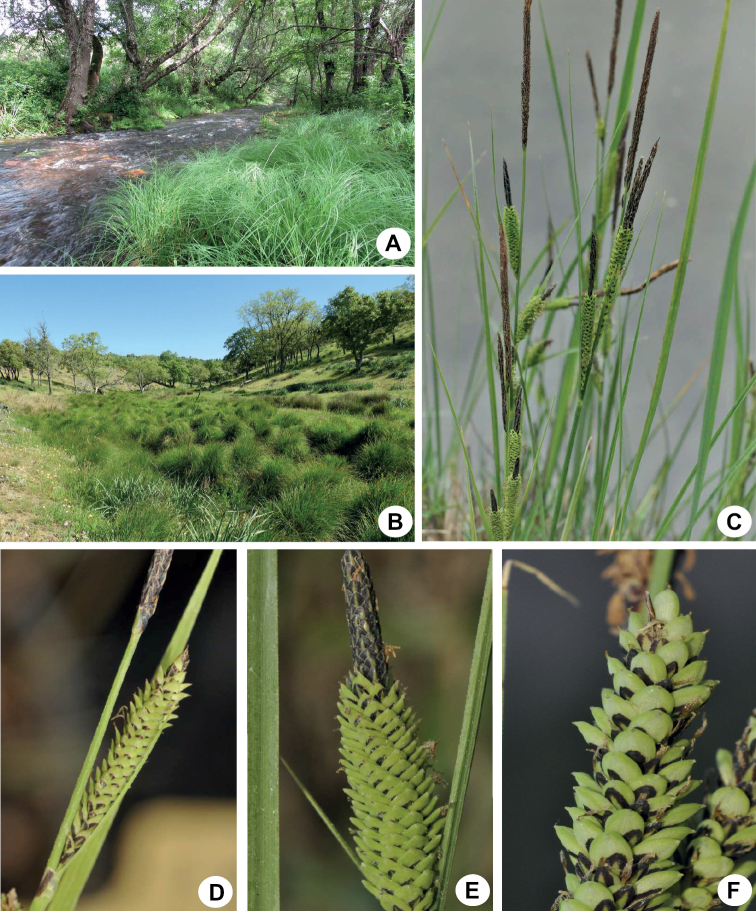
Representative photos of *Carexquixotiana***A** habitat (riparian forest with *Alnuslusitanica* and *Fraxinusangustifolia*: Ciudad Real, Solana del Pino, Robledillo River; 57SMB17, UPOS-8924; *C.quixotiana* plants are in the lower right corner) **B** habitat (stream in *Quercuspyrenaica* open forest: Ciudad Real, between El Viso del Marqués and San Lorenzo de Calatrava; 8CBB18, UPOS-16897; *C.quixotiana* are the tussocks along the stream) **C** inflorescence **D, E** detail of terminal male and lateral androgynous spikes **F** detail of lateral female spikes and utricles (Ciudad Real, Fuencaliente, Minas del Horcajo, 54SMB17, UPOS-8922).

During the preparation of Flora Iberica (Luceño and Jiménez-Mejías 2008) a set of problematic Carexsect.Phacocystis populations from Sierra Madrona (a northern subrange of Sierra Morena belonging to La Mancha) were tentatively assigned to *C.reuteriana* (Jiménez-Mejías et al. 2011), but with ambiguous affinities regarding the subspecies (C.reuterianasubsp.reuteriana and *mauritanica*; treated under *C.elata* in Flora Iberica, Luceño and Jiménez-Mejías 2008). A preliminary AFLP assessment (Jiménez-Mejías and Martín-Bravo, unpublished) associated with a study focusing on *C.reuteriana* (Benítez-Benítez et al. 2018) revealed that, surprisingly, the problematic populations were not as closely related to *C.reuteriana* as expected. Subsequently, we found several additional of these problematic populations in La Mancha. The detailed comparison of the material with other Iberian species of the group revealed superficial morphological affinities with *C.reuteriana* or *C.nigra*. However, its ecological preferences were confounding, since the problematic populations inhabited generally small streams, rivers and springs and wet meadows in marcescent and sclerophyllous *Quercus* forests (Fig. 2), while *C.reuteriana* grows exclusively in permanent streams, and *C.nigra* thrives at much higher altitudes in wet mountain meadows and bogs. Here we conduct a detailed systematic study of these La Mancha's problematic populations, including the comparison with other Iberian species within sect. Phacocystis, using a combined molecular, morphological and cytogenetic approach demonstrated to be useful for disentangling the complex taxonomy of this group (e.g., Jiménez-Mejías et al. 2011). Our objective was to elucidate the systematic status of these populations and to warrant their taxonomic recognition if required.

## ﻿Materials and methods

### ﻿Sampling

We collected specimens from 10 problematic populations from La Mancha region (Sierra Madrona and Montes de Toledo; Fig. 1) that were deposited at UPOS herbarium, with duplicates at GDA, JAEN, K, MA, NY, P and SALA (acronyms following Thiers 2023). A special sampling effort was conducted in Sierra Madrona, resulting in a representative sampling across the entire range (Fig. 1). Additional herbarium specimens were obtained from JAEN, MA, MACB and SALA (Suppl. material 1). Some of these specimens came from sites very close to the 10 mentioned populations (e.g., in the same watercourse) and/or had imprecise coordinates, so they were considered to belong to the same population, but 6 additional populations were identified (Suppl. material 1).

### ﻿Morphological study

Material from all 16 sampled populations (Suppl. material 1) was carefully compared with all the other sect. Phacocystis species present in the Western Palearctic using specialized literature (Schultze-Motel 1968; Nilsson 1985; Kukkonen 1998; Egorova 1999; Jermy et al. 2007; Dean and Ashton 2008; Luceño and Jiménez-Mejías 2008; Jiménez-Mejías et al. 2015), with emphasis on Iberian species and specifically the morphologically similar *C.nigra* and *C.reuteriana*. We examined the most important morphological characters for the taxonomy of Carexsect.Phacocystis: general habit, basal sheaths colour and form, stomata distribution on leaf faces, relative length of lowest bract/inflorescence, spike size and sex distribution, and utricle size, colour and indumentum. Measurements above 1 cm were taken with a standard 30 cm ruler, and those below 1 cm with an ocular micrometer (only for specimens from the 10 collected populations deposited at UPOS).

### ﻿Molecular and phylogenetic study

To tackle the phylogenetic placement of La Mancha’s problematic populations, we included these samples in the molecular phylogeny of sect. Phacocystis (Benítez-Benítez et al. 2021). We used the same markers as in that phylogeny: two nuclear ribosomal markers (ITS-ETS) and two plastid markers (*rpl*32‐*trn*L^UAG^ and *ycf*6‐*psb*M). The new sequenced material (Suppl. material 2, including Genbank accessions) was integrated with the complete singleton matrix (Benítez-Benítez et al. 2021) (Suppl. material 3), which provided the best phylogenetic resolution in that study. That matrix included 75% of the species of the section and 80% of the so-called Western Palearctic clade in particular (Benítez-Benítez et al. 2021). Inferred hybrid samples (e.g., *C.acuta*, *C.buekii*, *C.randalpina*, *C.salina*) were excluded from the complete singletons matrix to avoid deleterious topological effects as explained in Benítez-Benítez et al. (2021). The morphological and biogeographic affinities of La Mancha’s problematic populations clearly point to the Western Palearctic lineage as the best candidate for them to be placed in.

DNA extraction and sequence amplification followed Benítez-Benítez et al. (2021). All PCR products were sequenced by Macrogen (Madrid, Spain). Sequence chromatograms were edited using GENEIOUS v.11.0.2 (Biomatters Ltd., Auckland, New Zealand). The sequences were aligned with MUSCLE v.5 (Edgar 2004). We carried out a scaffolding strategy following Jiménez-Mejías et al. (2016) and Benítez-Benítez et al. (2021) methodologies. First, we compiled all accessions containing both nuclear sequences (ITS and ETS) and built a nrDNA reference tree using maximum likelihood (ML) with RAXML (Stamatakis 2014; 100 bootstrap replicates). The resulting tree was used to build a query tree placing all the remaining accessions through the Evolutionary Placement Algorithm (EPA; Berger et al. 2011), also implemented in RAXML. Branch support for the query tree was calculated using the non-parametric Shimodaira-Hasegawa (SH) from approximate likelihood ratio test (SH-aLRT support; Guindon et al. 2010; Anisimova et al. 2011). All analyses were implemented in CIPRES Science Gateway (Miller et al. 2010).

To explore the systematic relationships of La Mancha’s problematic populations at a finer scale and with respect to the other Iberian representatives of sect. Phacocystis, a statistical parsimony analysis of plastid haplotypes was conducted with TCS v. 1.2.1 (Clement et al. 2000). We included only those accessions from specimens from the Iberian Peninsula that contained both plastid *rpl*32‐*trn*L^UAG^ and *ycf*6‐*psb*M regions, except for *C.acuta* and *C.buekii* as explained above. To calculate the most parsimonious haplotype network we set a 95% parsimony connection limit for the minimum number of mutations differentiating the haplotypes. Gaps in sequences were coded as a fifth character.

### ﻿Cytogenetic study

Chromosome counts for five La Mancha’s problematic populations (Suppl. material 1) were performed. Anthers, from young male flowers, were fixed during meiosis following the method by Luceño (1988). The obtained chromosome numbers and meiotic configurations were compared with those of closely related species, since cytogenetics have been demonstrated to have a strong diagnostic potential in *Carex*, including sect. Phacocystis (Jiménez-Mejías et al. 2011).

### ﻿Conservation Assessment

Following the taxonomic recognition of La Mancha’s problematic populations (see below), we evaluated their conservation status at the global level following criteria, categories, and guidelines from IUCN (2017). Area of occupancy (AOO) and extent of occurrence (EOO) were calculated using the GeoCAT tool (Bachman et al. 2011) based on the 16 studied populations (see morphological study).

## ﻿Results

### ﻿Morphological study

The detailed examination of diagnostic morphological characters for La Mancha’s problematic populations and its comparison with closely related species revealed qualitative and quantitative morphological differences regarding the other Iberian taxa of sect. Phacocystis (Table 1). In particular, all character states of La Mancha’s problematic populations were distinct from at least one of the other Iberian taxon studied.

**Table 1.** Comparison of main morphological diagnostic characters, chromosome numbers, and habitat (on the Iberian Peninsula) among *C.quixotiana* and all the other Iberian Carexsect.Phacocystis taxa. Measurements from the other taxa have been taken from Luceño and Jiménez-Mejías (2008).

	*C.quixotiana*	*C.acuta*	*C.cespitosa*	C.elatasubsp.elata	*C.nigra*		*C.reuteriana*		*C.trinervis*
					subsp. nigra	subsp. intricata	subsp. reuteriana	subsp. mauritanica	
Habit	From tussock-forming to rhizomes elongated	From more or less caespitose to rhizomes elongated	Tussock-forming	From tussock-forming to laxly caespitose	Rhizomes elongated		Tussock-forming		Rhizomes elongated
Basal sheaths (at the base of fertile stems)	Scale-like, sometimes elongated, creamy-yellow, rarely reddish-brown	Culm bases bearing brown old-leaf remains, without conspicuous scale-like basal sheaths	Scale-like, dark purple	Scale-like to elongated, creamy-yellow	Culm bases with or without conspicuous scale-like basal sheaths, dark brown when present		Scale-like, orange to reddish-brown		Culm bases bearing straw-colored old-leaf remains, without conspicuous scale-like basal sheaths
Leaf section	Flat to keeled	Flat	Flat to keeled	Flat to keeled	Flat to keeled		Flat to keeled		Strongly plicate to canaliculate
Leaf width (mm)	(1.8)2–3.2(4.7)	3–5(7)	2–4	3–6(7)	3–6(10)		(3)3.5–5.5(6)	(3)4–8(10)	(0.5)1.5–2.5(3)
Stomata distribution	Amphistomatic	Hypostomatic	Hypostomatic	Hypostomatic	Epistomatic	Amphistomatic	Hypostomatic		Amphistomatic
Relative lowermost bract-inflorescence length	Longer to shorter than the inflorescence	Longer than the inflorescence	Shorter than the inflorescence, rarely equalling it	Shorter than the inflorescence, rarely equalling or exceeding it	Equalling to slightly shorter than the inflorescence, rarely exceeding it		Equalling the inflorescence		Longer than the inflorescence
Terminal (male) spikes number	(1)2–3	2–4	1	1–2	1(2)		1(2)	(1)2–4	(1)2–3
Terminal (male) spike length	(15)20–60(85)	(15)20–50(60)	(12)15–30	25–80	(5)10–30		(15)20–60(70)	(10)20–70	(15)25–40
Lateral androgynous spikes	(0)1–3(4)	1–2	0	0–3(4)	(0)1		0–3(6)	(0)1–5	2–4
Lateral female spikes	0–2(3)	2–4	1–2(3)	0–2(3)	2–4		0–3(4)	0(1)	2–4
Utricle nerves	Nerved	Nerved	Nerveless	Nerved	Nerved		Nerved		Nerved
Utricle indumentum	With high whitish papillae on the upper half or towards the apex, sometimes aculeolate at the apex	Almost entirely covered with high papillae, somewhat inflated	With whitish high papillae towards the apex	With whitish high papillae, at least towards the apex, not inflated	Covered with high papillae at least the upper half		Smooth, very rarely with some scattered, low papillae towards the apex		Covered with low papillae
Chromosome number (2n)	82–83	84–86	78–80	74–80	(80)82–88(92?)		73–76	(72)74–76	82–85
Habitat (Iberian Peninsula)	Small streams, rivers and springs in riparian forests (*Alnuslusitanica*, *Fraxinusangustifolia*, *Salix* spp.), humid meadows, in marcescent and sclerophyllous *Quercus* forests, on siliceous bedrock substrate	River shores, usually in deciduous forests, without clear edaphic preferences regarding bedrock substrate	River shores in deciduous forest, on granitic substrates	River shores (or lakes) in diverse types of vegetation, on calcareous bedrock substrates, rarely on siliceous ones, also on coastal swamps	Montane-alpine wet meadows, bogs, and swamps, also in river and lake shores, in diverse types of vegetation and without clear edaphic preferences regarding bedrock substrate		Stream and river shores in diverse types of vegetation, usually on siliceous bedrock substrates, rarely on calcareous ones	Stream and river shores in riparian forests, in marcescent and sclerophyllous *Quercus* forests, without clear edaphic preferences regarding bedrock substrate	Sandy coastal swamps
Altitude (m)	400–800	0–1300	10–40	10–1750	1000–3300		300–1900	20–650	0–30

### ﻿Molecular study

**Figure 3.**
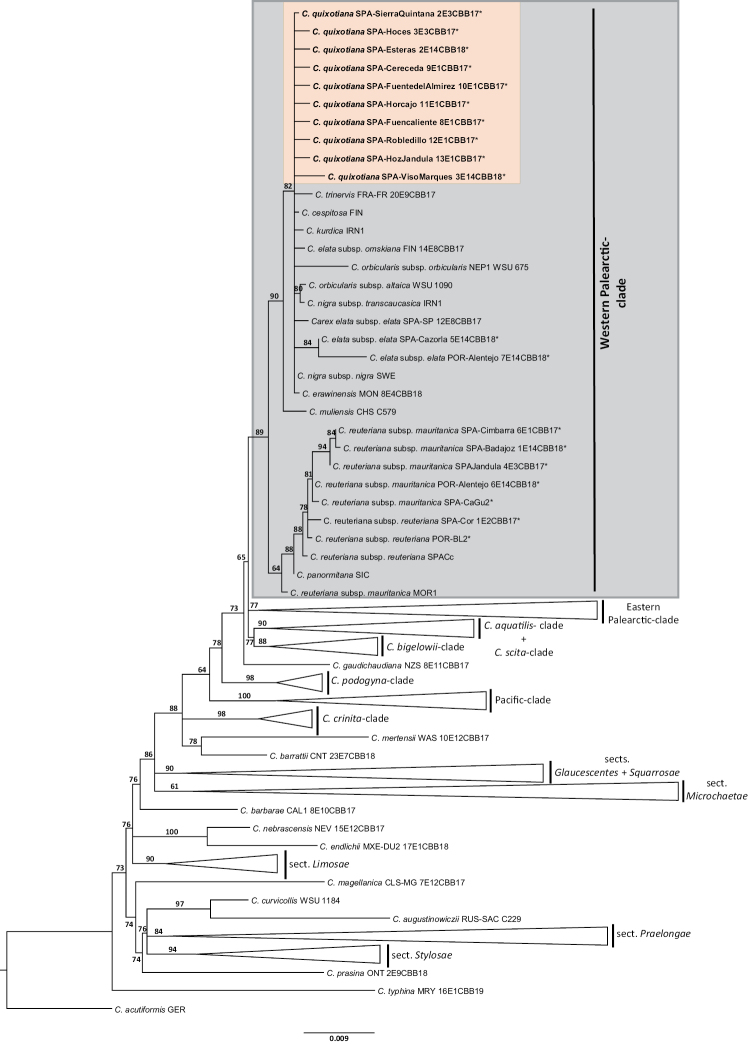
Phylogram of sect. Phacocystis s.l. based on the maximum likelihood phylogenetic reconstruction of the complete singletons tree (ITS, ETS, *rpl*32‐*trn*L^UAG^, *ycf*6‐*psb*M) by Benítez-Benítez et al. (2021). SH supports > 60 are given above branches. Tip codes are included in Suppl. material 3. The samples with asterisk (*) have been newly sequenced for this study. The orange highlighted populations represent La Mancha’s problematic samples herein described as *Carexquixotiana*.

The query tree built (see Suppl. material 4) using the singletons matrix from Benítez-Benítez et al. (2021) recovered a topology equivalent to that found in the reference paper. The Western Palearctic clade was relatively well-supported (SH=89) and arranged in two main clades (Fig. 3): one containing *C.reuteriana* and the closely related *C.panormitana* (marginally supported, SH=64), and another with the rest of the species of the clade (SH=90). La Mancha’s problematic populations were placed unresolved among the species of this latter clade.

The statistical parsimony analysis revealed 14 different haplotypes (Suppl. materials 5, 6). La Mancha’s problematic populations displayed an exclusive haplotype, not shared with any of the other Iberian species. The most closely related haplotype was that displayed by the Sierra Nevada populations of *C.nigra*, separated by only one mutation.

### ﻿Cytogenetic study

**Figure 4.**
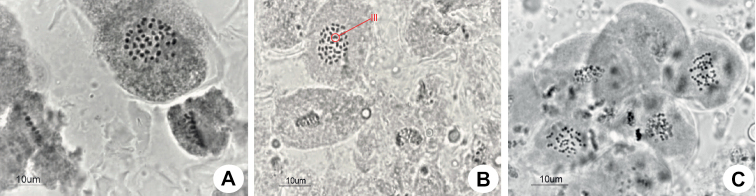
**A** regular meiotic configuration of *Carexquixotiana* in Metaphase I (2n=82) **B** meiotic configuration showing a trivalent in Metaphase I (2n=40^II^ + 1 ^III^=83) **C** pollen grain mitosis of the irregular cytotype in which two cells are visible (n=41 and n=42).

The studied meiotic plates, representing five different La Mancha’s problematic populations (Suppl. material 1), mostly displayed 82 chromosomes, with regular pairing (Fig. 4). Interestingly, the population 59SMB17 also showed 40^II^ + 1^III^ in Metaphase I, and n=41, 42 in pollen grain mitosis (from 30 counted plates, 16 showed n=41 and 14 n=42). Therefore, the inferred diploid chromosome numbers were 2n=82, 83 (Fig. 4).

## ﻿Discussion

Different sources of evidence (morphological, cytogenetic, molecular) support the systematic distinctiveness of the studied La Mancha’s problematic populations (Table 1, Fig. 3, Suppl. material 6). The greater morphological affinities of these populations are with *C.reuteriana* and *C.nigra*, however they show clear-cut morphological differences (Table 1). La Mancha’s populations differ from these two species by leaf width and basal sheath colour. In addition, it differs from *C.reuteriana* by leaf stomata distribution, utricle indumentum and chromosome number (*C.reuteriana* (72)73–76 vs. *C.quixotiana* 82–83), and from *C.nigra* by the terminal male spike length. In addition to the appearance, the ecology of *C.nigra* in Sierra Nevada is fairly distinct as it is a dwarf sedge that forms dense tufts at habitats often above the timberline (from 1000 m and up to 3300 m), while La Mancha’s populations are medium-size herbs that grow in low-medium elevation in riparian vegetation within *Quercus* forests.

Remarkably, the DNA markers used in the phylogenetic reconstructions showed that La Mancha’s problematic populations do not group with *C.reuteriana*, to which they had been traditionally assigned (Luceño and Jiménez-Mejías 2008), while they are more related to *C.nigra* s.l. (Fig. 3). In addition, the haplotype network pointed to more genetic affinities with the populations of *C.nigra* from Sierra Nevada (Suppl. material 6), which are ca. 150 km south of La Mancha, than with *C.reuteriana*.

According to the evidence presented, La Mancha’s problematic populations warrant taxonomic recognition, so we proceed to describe them as a new species.

### ﻿Taxonomic treatment

#### ﻿ Carex quixotiana

Taxon classificationPlantaePoalesCyperaceae

Ben.Benítez, Martín-Bravo, Luceño & Jim.Mejías sp. nov.

CF3E8E18-33F3-5708-B8CC-92F6C720E04C

urn:lsid:ipni.org:names:77315699-1

##### Diagnosis.

Similar in appearance to *C.reuteriana* Boiss. & Reut., from which it differs by the creamy-yellow, rarely reddish-brown basal sheaths (vs. orange to reddish-brown), amphistomatic leaves (vs. hypostomatic), and utricles with high papillae (vs. smooth or rarely with low papillae). It is also similar to *C.nigra* (L.) Reichard, from which it can be distinguished also by the creamy-yellow, rarely reddish-brown basal sheaths (vs. absent or dark brown when present), narrower leaves (1.8)2–3.2(4.7) mm (vs. 3–6(10) mm), and longer terminal male spike (18)20–60(85) mm (vs. (5)10–30 mm).

##### Type.

**Spain.** Ciudad Real: Fuencaliente, Azor stream recreational area, stream edges in *Quercusfaginea* forests, 733m, 38.44906944, -4.327163889, 10 May 2017, S. Martín-Bravo & C. Benítez-Benítez 41SMB17 (holotype!: UPOS-8925, 41SMB17(5); isotypes!: GDA, JAEN, K, MA, NY, P, SALA and UPOS).

##### Selected material examined

**(paratypes): Spain.** Ciudad Real: Fuencaliente, Cereceda stream, with *Alnuslusitanica*, 695m, 38.42363889, -4.297472222, 10 May 2017, S. Martín-Bravo & C. Benítez-Benítez 47SMB17 (UPOS-8927); Fuente del Almírez, puddled meadows in *Quercuspyrenaica* forests, 800 m, 38.47152778, -4.344888889, 10 May 2017, S. Martín-Bravo & C. Benítez-Benítez 48SMB17 (UPOS-8926); Minas del Horcajo, gorge of Nacedero stream with *Salix* sp., 729 m, 38.51397222, -4.445750000, 11 May 2017, S. Martín-Bravo & C. Benítez-Benítez 54SMB17 (UPOS-8922); Solana del Pino, Robledillo River, riparian forests with *Alnuslusitanica* and *Fraxinusangustifolia*, 453 m, 38.41783333, -4.003388889, 11 May 2017, S. Martín-Bravo & C. Benítez-Benítez 57SMB17 (UPOS-8924); Solanilla del Tamaral, gorge of Jandula River (Hoz del Jándula) with *Alnuslusitanica*, 392 m, 38.39186111, -3.96333333, 11 May 2017, S. Martín-Bravo & C. Benítez-Benítez 59SMB17 (UPOS-8923); Valdemancos de Esteras, riverside of Esteras River with *Fraxinusangustifolia* and *Salix* sp., 453 m, 38.90733333, -4.794222222, 15 May 2018, C. Benítez-Benítez & S. Martín-Bravo 6CBB18 (UPOS-16896); Between Viso del Marqués and San Lorenzo de Calatrava, riverside of Ballesteros stream, in open forest of *Quercuspyrenaica* and *Salix* sp., 872 m, 38.44450000, -3.740444444, 16 May 2018, C. Benítez-Benítez & S. Martín-Bravo 8CBB18 (UPOS-16897); Puebla de Don Rodrigo, Sala del Halconcillo stream, 559 m, 39.10527778, -4.744722222, 15 June 2022, M. Sanz-Arnal, P. García-Moro & P. Jiménez-Mejías 13MSA22 (UPOS-16898); Horcajo de los Montes, Chorrera de Horcajo, 642 m, 39.36111111, -4.614999999, 15 June 2022, M. Sanz-Arnal, P. García-Moro & P. Jiménez-Mejías 16MSA22 (UPOS-16899); Viso del Marqués, Las Hoces, 780 m, 38.42303569, -3.721722958, 16 May 1991, C. Fernández García-Rojo (JAEN-914254); Puebla de Don Rodrigo, Río Frío mountain range near a birch forest, 600 m, 39.08243815, -4.503007649, 9 May 1992, Carrasco, Garrido & Martín-Blanco (MACB-68849); Solana del Pino, valley of Nacedero stream, 590 m, 38.482505309, -4.169451970, 26 April 1997, R. García Río (MA-596319); Hinojosas, valley of Montoro River with *Alnusglutinosa*, Cervigón, 700 m, 38.491048629, -4.215454806, 5 May 1997, R. García Río (MA-596320); Almodóvar del Campo, Guadalmez River with *Alnusglutinosa*, 510 m, 38.513064049, -4.628724543, 4 July 1997, R. García Río (MA-596324). Jaén: Andújar, Sierra Quintana, Valmayor River, 570 m, 38.37460677, -4.144817294, 27 June 1985, E. Cano & C. Fernández García-Rojo (JAEN-855238).

##### Other material.

(see Suppl. material 1).

##### Morphological description.

***Rhizomes*** from dense and tussock-forming to elongated. ***Stems*** (48)60–80(95) cm long, (0.8)1–1.2(1.3) mm wide below the inflorescence, sharply trigonous, smooth for most of its length, densely scabrid above. ***Basal sheaths*** scale-like, sometimes elongated, creamy-yellow, rarely reddish-brown, coriaceous. ***Leaves*** (1.8)2–3.2(4.7) mm wide, pale green to bluish-green, amphistomatic, flat to keeled, usually shorter or equalling the stems, antrorsely scabrid on the margins, especially distally, and on the mid-vein on the abaxial side to the apex; ***ligule*** (2)3–7(10) mm long, usually more than twice as long as wide, apex acute to obtuse, rarely rounded or truncate, hyaline, the margins brownish to orange-brown. ***Inflorescence*** (9)12–19(22) cm long, all spikes erect, exceptionally the lowermost slightly nodding. Lowest bract leaf-like, very rarely setaceous, longer to shorter than the inflorescence, sheathless, with hyaline, pale brownish to dark purplish-brown auricles at its base. Terminal ***male spikes*** (1)2–3; the uppermost spike (15)20–60(85) × 2–3.5 mm, subsessile to long pedunculate, oblong-cylindrical to narrowly fusiform, densely flowered; subterminal male spikes (0)1–2, (5)10–30(33) × (1)1.5–2.3(2.6) mm, similar in outline to the terminal one, basally overlapping with it or with an internode up to 20 mm. Lateral spikes female or androgynous with the flowers spirally arranged; ***female spikes*** 0–2(3), (19)25–50(79) × (2)2.8–4(5.2) mm, cylindrical, densely to more or less laxly flowered proximally; ***androgynous spikes*** (0)1–3(4), (9)20–50(62) × (1)1.7–2.7(5) mm, with the male portion (1)3–15(50) mm long and the female one (6)10–40(50) mm long. ***Male glumes*** (1.9)2.3–3.9(4.1) × (0.6)0.7–1.2(1.4) mm, oblong to obovate-oblong, apex rounded, usually dark purplish brown, with a green, 1-veined central band, with or without whitish hyaline margins, sometimes also becoming hyaline towards the base. ***Female glumes*** 1.3–2.3(2.5) × (0.6)0.7–0.9(1) mm, lanceolate to ovate, rarely elliptical, apex obtuse, acute or mucronate, usually shorter and narrower than the utricles, very rarely shortly exceeding them, dark purplish brown, rarely pale brown, with a green, 1–3 veined central band, with or without whitish hyaline margins, mainly in the distal part. ***Utricles*** (1.8)2–2.7(3) × (1.1)1.3–1.8(2) mm, plano-convex, widely elliptical to almost suborbicular, green to straw-coloured, distally whitish, sometimes purplish-dotted or purplish-tinged towards the apex, with high, whitish papillae on the upper half or towards the apex, sometimes also aculeolate at the upper margins, faintly to conspicuously nerved, shortly stipitate, more or less abruptly contracted into a short, cylindrical, truncate, more rarely emarginate beak (0)0.1–0.2(0.3) mm long, whitish, sometimes brown-tinged. ***Achenes*** 1.5–2.1 × (1.1)1.3–1.5(1.7) mm, widely elliptical to suborbicular, straw-coloured to pale brown, biconvex, more or less stipitate; style base terete to slightly conical, up to 0.3 mm. Stigmas 2.

##### Distribution.

(Fig. 1) Endemic to South-Central Spain (Ciudad Real and marginally Jaén provinces). So far known from 16 populations (Suppl. material 1), mostly located in the Sierra Madrona range, but also extending north reaching the southern foothills of the Montes de Toledo range. Since it is a medium-size sedge rarely collected, there might be additional populations in these areas.

##### Habitat.

Small streams, rivers and springs in riparian forests (*Alnuslusitanica*, *Fraxinusangustifolia*, *Salix* spp.), and humid meadows, in marcescent and sclerophyllous *Quercus* forests, on siliceous bedrock substrate. 400–800 m.

##### Phenology.

(April) May-June (July).

##### Chromosome number.

2n=82, 83.

##### Iconography.

Fig. 5.

**Figure 5.**
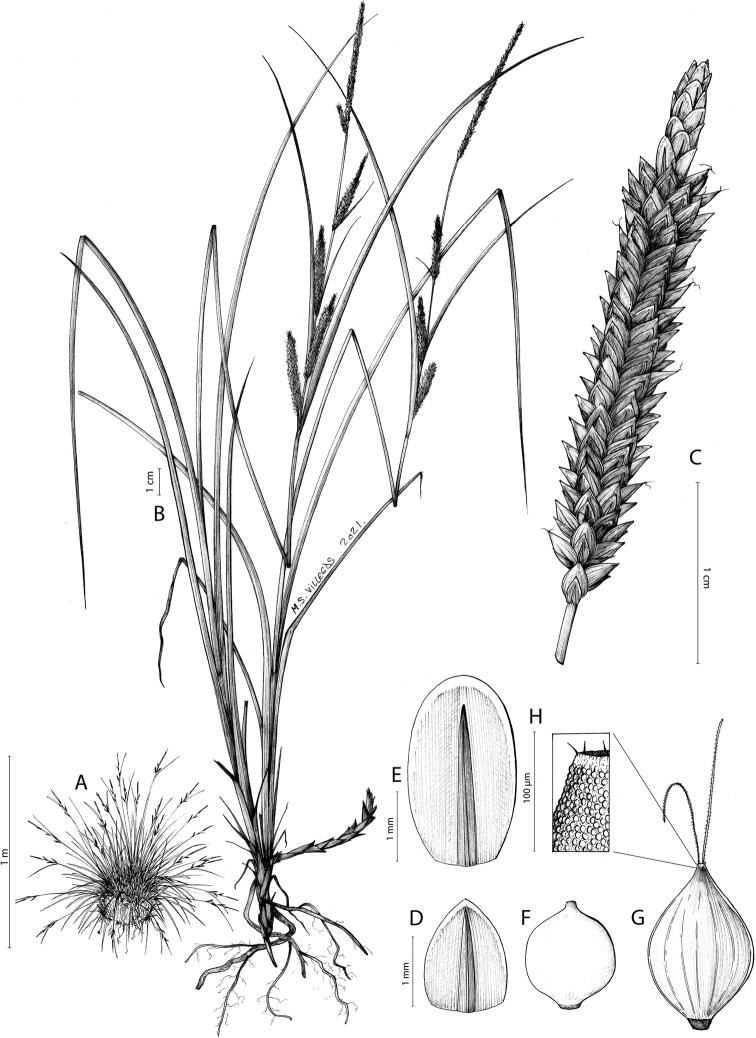
Analytical drawing of *Carexquixotiana* (Spain, Ciudad Real, Sierra Madrona) **A** habit **B** plant **C** androgynous spike **D** female glume **E** male glume **F** achene **G** utricle **H** details of papillae in the upper part of the utricle. Drawing by M. Sánchez-Villegas.

##### Conservation status.

So far known from 16 populations (subpopulations according to IUCN; Fig. 1), deduced from the studied material (Suppl. material 1). This implies a relatively restricted distribution range enclosed in an extent of occurrence (EOO) of 4.920 km^2^, and an area of occupancy (AOO) of just 64 km^2^. This would point to the application of criteria B1 and B2 of the Endangered (EN) category (threshold of 5.000 km^2^ and 500 km^2^ for EOO and AOO, respectively; IUCN 2017). The species’ overall demographic tendency and number of mature individuals are unknown. In addition, the number of locations (16; n > 5) prevents the application of EN category since two conditions of criteria B must be fulfilled. Some populations are located in protected land (see below) so they should not be likely submitted to plausible threats in the short term (but see García Río 2007). With the currently available data, and taking into account the restricted EOO and AOO, we hypothesize that *C.quixotiana*’s formal IUCN conservation category at the global level would be Data Defficient (DD). As it is an Iberian endemic with a relatively small number of populations and distribution range, it would benefit from legal protection and inclusion in in-situ/ex-situ conservation programmes, at least at the regional level (Castilla-La Mancha, Andalucía).

##### Etymology.

The species epithet, *quixotiana* (pronounced *kee·how·tee·a·na* in English) is derived from Miguel de Cervantes’s (1547–1616) masterpiece Don Quixote (de Cervantes 1605, 1615), globally considered one of the best works in the history of literature, and whose number of editions and translations is only surpassed by the Bible. The setting of Don Quixote is La Mancha, the region of Spain where almost all populations of *Carexquixotiana* occur. We would like this epithet to serve as a double tribute: (1) First to Cervantes and his novel Don Quixote, flagship of Spanish culture. And (2) To Pedro Jiménez-Mejías’s father, Pedro Jiménez Gilabert, an enthusiastic reader who always enjoys reading Don Quixote above all other books, and who always transmitted Pedro’s curiosity and love for nature. In contrast to the first words in Don Quixote, (“En un lugar de La Mancha, de cuyo nombre no quiero acordarme (...)” [In a village of La Mancha, the name of which I have no desire to call to mind (…)]), we desire to remember and commemorate the dedication of this epithet we are coining.

##### Systematic notes.

*Carexquixotiana* populations have been consistently assigned to *C.reuteriana*. Local floristic studies have predominantly identified them as C.reuterianasubsp.reuteriana (e.g., García Río 2004, 2007; Martín Blanco and Carrasco 2005; Fernández García-Rojo 2015; Fernández García-Rojo and Salazar-Mendías 2019), but also as C.reuterianasubsp.mauritanica (García Río 1999, 2004). Specialized taxonomic treatments have considered the populations as morphologically intermediate between the two *C.reuteriana* subspecies, which has been attributed to hybridisation in a putative contact zone between the two *C.reuteriana* subspecies (Luceño and Jiménez-Mejías 2008).

These problematic populations clearly illustrate the taxonomic complexity of sect. Phacocystis. Their overall morphological appearance and ecological preferences suggested that the populations were conspecific with *C.reuteriana* (Luceño and Jiménez-Mejías 2008). On the other hand, phylogenetic relationships (Fig. 2) and the relatively high chromosome number (see below) indicate closer genetic affinity with *C.nigra*. These misleading patterns denote the necessity of integrative approaches comprising different data sources to unmask complex systematic scenarios. Further phylogenomic studies based on High-Throughput-Sequencing techniques like GBS might provide a greater number of loci and therefore might help to shed more light on the systematic placement of *C.quixotiana* (Benítez-Benítez et al. unpublished data).

The chromosome number has been used as a biosystematic tool for sect. Phacocystis in the Iberian Peninsula (Luceño and Aedo 1994; Luceño and Jiménez-Mejías 2008). Our chromosome counts indicate that *C.quixotiana* (2n=82–83) has a greater cytogenetic affinity with *C.nigra* (2n=(80)82–86) than with *C.reuteriana* (2n=(72)73–76).

##### Biogeographic and conservation issues.

Considering the new species described here, the Iberian Peninsula has 12 endemic *Carex* taxa (*C.asturica* Boiss., C.camposiiBoiss. & Reut.subsp.camposii, C.camposiisubsp.tejedensis R. Sánchez-Villegas, M.Escudero & Luceño, *C.caudata* (Kük.) Pereda & Laínz, *C.durieui* Steud. & Kunze, *C.furva* Webb, *C.lainzii* Luceño, E.Rico & T.Romero, *C.lucennoiberica* Maguilla & M. Escudero, *C.nevadensis* Boiss. & Reut., *C.quixotiana*, C.reuterianassp.reuteriana and *C.rorulenta* Porta). However, this may appear as a relatively small number of endemics in proportion to the total number of *Carex* Iberian native taxa (108; Luceño et al. unpublished data), especially when compared with other species-rich genera (e.g., *Alchemilla*, *Armeria*, *Centaurea*, *Limonium*, *Teucrium*) with much larger numbers of Iberian endemics (>40 each; Aedo et al. 2017; Buira et al. 2020). This highlights the novelty of the description of an additional Iberian endemic in *Carex*. Indeed, *C.quixotiana*, with a highly restricted distribution in southern-central Spain, would be the first Iberian endemic species belonging to sect. Phacocystis, although one subspecies (C.reuterianassp.reuteriana) is also endemic to C-NW parts of the Iberian Peninsula. Other species of sect. Phacocystis with limited distribution range in the Mediterranean basin are C.reuterianassp.mauritanica (S Iberian Peninsula-NW Africa), C.nigrassp.intricata (Western Mediterranean mountains), and *C.panormitana* Guss. (Sicily, Sardinia and Tunisia).

As explained above, known populations of *C.quixotiana* are mostly located in La Mancha limits, in Sierra Madrona and, to a lesser extent, southern Montes de Toledo. Sierra Madrona is one of the secondary mountain ranges within the larger Sierra Morena range, which stretches for about 450 kms from W to E across the S Iberian Peninsula, separating the southern half of the Central Plateau from the Guadalquivir Valley. Sierra Madrona runs almost in parallel (NW–SE) along the northern side of the main range for about 80 kms. It includes the highest altitudes of all Sierra Morena (Bañuela peak, 1332 m). As the whole Sierra Morena, it is mainly composed by old Paleozoic siliceous rocks, especially quartzites. On the other hand, Montes de Toledo is a mountain range entirely belonging to the southern half of the Central Plateau, separating the Tajo and Guadiana River basins. It stretches for about 350 kms from E to W and is composed by quartzite ridges of relatively uniform elevation, around 1400 m, and a generally eroded relief (Muñoz Jiménez 1976). The immediate foothill landscapes are highly anthropised, with the vast majority of La Mancha dedicated to non-irrigated crops, mostly cereal (wheat, barley and oat), and vineyards.

Sierra Madrona is currently protected as a natural park established in 2011 (Valle de Alcudia and Sierra Madrona). Several restricted plant endemics from Sierra Morena are found in this range and its surroundings (i.e., *Armeriapauana* (Bernis) Nieto Fel., *Coincyalongirostra* (Boiss.) Greuter and Burdet; García Río 2004). It also includes many different habitats protected at European level (Directive “Habitats” 92/43/CEE; see García Río 2004, 2007), including some considered of high conservation priority. *Carexquixotiana* habitats in Sierra Madrona, although included in a protected area, are not free of threats, so their biodiversity has been considered as highly valuable and sensitive (García Río 2007). They include populations of other endangered and protected plant species at regional (e.g., *Ericatetralix* L., *Droserarotundifolia* L.) or even at national level (e.g., *Myricagale* L., *Narcissus muñozii-garmendiae* Fern.Casas).

The other known populations of *C.quixotiana* occur mostly in rivers and creeks in the southern foothills of Montes de Toledo in W Ciudad Real province. These populations are located within or close to remarkable habitats, such as relictual peatbogs (e.g., Bonales de Puebla de Don Rodrigo) and birch forests (e.g., Abedular de Ríofrío), which are on protected land and considered to be of extraordinary conservation value. In addition, *C.quixotiana* marginally reaches NE Jaén province in Sierra Quintana, a small southern subrange of Sierra Madrona province (Cano-Carmona and Valle-Tendero 1996), which separates Castilla-La Mancha from Andalucía (Fig. 1). Sierra Madrona and Montes de Toledo are considered biogeographically close (García Río 2007). Interestingly, C.reuterianasubsp.reuteriana occurs in northern parts of Montes de Toledo, whereas C.reuterianasubsp.mauritanica is widespread in the main Sierra Morena range, therefore flanking C.quixotiana's distribution (Luceño and Jiménez-Mejías 2008; see maps in Benítez-Benítez et al. 2018). It would be interesting to precisely delimit these species’ range and to explore potential contact zones in detail in order to investigate the possible co-occurrence and/or hybridisation processes.

### ﻿Identification key to the Iberian taxa of CarexsectionPhacocystis

**Table:** 

1	Leaves strongly plicate to canaliculate, rigid, up to 2.5(3) mm wide; stems obtusely trigonous; plants bluish-green	***C.trinervis***
–	Leaves flat to carinate, soft to medium rigid, (1.8)2–8(10) mm wide; stems sharply trigonous; plants green to bluish-green	**2**
2	Utricles not veined; basal sheaths dark purple	***C.cespitosa***
–	Utricles faintly to prominently veined; basal sheaths yellowish-brown to reddish-brown	**3**
3	Utricles smooth, rarely with some scattered, green, low papillae	**4**
–	Utricles densely papillose, at least on the apex, with whitish, more or less high papillae	**5**
4	Male spike usually solitary, rarely 2; lowest spikes usually female; leaves up to 6 mm wide	**C.reuterianasubsp.reuteriana**
–	Male spikes (1)2–4; lowest spikes usually androgynous; leaves (3)4–8(10) mm wide	**C.reuterianasubsp.mauritanica**
5	Leaves epistomatic or amphistomatic	**6**
–	Leaves hypostomatic	**8**
6	Male spikes (1)2–3, the terminal one (15)20–60(85) mm long; leaves (1.8)2–3.2(4.7) mm wide; plants usually tussock-forming; basal sheaths scale-like, sometimes elongated, creamy yellow, rarely reddish-brown	***C.quixotiana***
–	Male spike solitary, rarely 2, the terminal one (5)10–30 mm long; leaves 3–6(10) mm wide; plants rarely tussock-forming; scale-like basal sheaths absent or dark brown when present	**7**
7	Leaves densely stomatic on both sides (amphistomatic)	***C.nigra* subsp**. ***intricata***
–	Leaves epistomatic or densely stomatic on the upper surface and with a few scattered stomata on the lower surface	**C.nigrasubsp.nigra**
8	Lowest bract much longer than the inflorescence; utricles strongly biconvex, somewhat inflated; culm bases bearing brown old leaf remains, without conspicuous scale-like basal sheaths	***C.acuta***
–	Lowest bract always shorter than the inflorescence; utricles plano-convex to slightly biconvex, not inflated; basal sheaths scale-like to elongated, creamy-yellow	**C.elatasubsp.elata**

## ﻿Conclusion

Integrative approaches based on different sources of evidence are required to unveil complex systematic scenarios. Our study of some problematic Iberian populations belonging to the taxonomically difficult sect. Phacocystis revealed that they display a congruent set of diagnostic morphological, molecular, ecological and cytogenetic characters that allow their distinction with respect to close relatives (e.g., *C.reuteriana*, *C.nigra*). Therefore, we here described a new species to science (*C.quixotiana*) for these populations, which is endemic to the Iberian Peninsula.

## Supplementary Material

XML Treatment for Carex quixotiana

## Supplementary materials

Supplementary material 1 List of studied material of *C.quixotiana*, including the 16 known populations highlighted in red Data type occurrence data (excel document) https://binary.pensoft.net/file/824891This dataset is made available under the Open Database License (http://opendatacommons.org/licenses/odbl/1.0/). The Open Database License (ODbL) is a license agreement intended to allow users to freely share, modify, and use this Dataset while maintaining this same freedom for others, provided that the original source and author(s) are credited.Carmen Benítez-Benítez, Pedro Jiménez-Mejías, Modesto Luceño, Santiago Martín-Bravo

Supplementary material 2 List of specimens newly sequenced in the present study Data type Occurrence data (excel document) Explanation note Including taxon, sample code, locality, voucher and/or herbarium number, and NCBI accession numbers for each molecular marker (ITS, ETS, *rpl*32‐*trn*LUAG, *ycf*6‐*psb*M). https://binary.pensoft.net/file/824892This dataset is made available under the Open Database License (http://opendatacommons.org/licenses/odbl/1.0/). The Open Database License (ODbL) is a license agreement intended to allow users to freely share, modify, and use this Dataset while maintaining this same freedom for others, provided that the original source and author(s) are credited.Carmen Benítez-Benítez, Pedro Jiménez-Mejías, Modesto Luceño, Santiago Martín-Bravo

Supplementary material 3 List of studied material, with their accession numbers included, which were previously published by Benítez-Benítez et al. (2021) Data type occurrence data (excel document) Explanation note Tip codes with “spm” denote samples previously published by Global Carex Group works ([Bibr B31][Bibr B41]). https://binary.pensoft.net/file/824893This dataset is made available under the Open Database License (http://opendatacommons.org/licenses/odbl/1.0/). The Open Database License (ODbL) is a license agreement intended to allow users to freely share, modify, and use this Dataset while maintaining this same freedom for others, provided that the original source and author(s) are credited.Carmen Benítez-Benítez, Pedro Jiménez-Mejías, Modesto Luceño, Santiago Martín-Bravo

Supplementary material 4 Cladogram of sect. Phacocystis, including newly sampled sequences Data type phylogenetic tree (PDF file) Explanation note The cladogram is based on the maximum likelihood phylogenetic reconstruction of complete singleton’s tree (ITS, ETS, *rpl32-trn*L, *ycf6-psb*M) following a scaffolding approach previously implemented by Benítez-Benítez et al. (2021). SH supports > 60 are given above branches. https://binary.pensoft.net/file/824894This dataset is made available under the Open Database License (http://opendatacommons.org/licenses/odbl/1.0/). The Open Database License (ODbL) is a license agreement intended to allow users to freely share, modify, and use this Dataset while maintaining this same freedom for others, provided that the original source and author(s) are credited.Carmen Benítez-Benítez, Pedro Jiménez-Mejías, Modesto Luceño, Santiago Martín-Bravo

Supplementary material 5 List of studied material for haplotype network analysis, displaying the corresponding haplotype number obtained for each sample Data type table (excel document) https://binary.pensoft.net/file/824895This dataset is made available under the Open Database License (http://opendatacommons.org/licenses/odbl/1.0/). The Open Database License (ODbL) is a license agreement intended to allow users to freely share, modify, and use this Dataset while maintaining this same freedom for others, provided that the original source and author(s) are credited.Carmen Benítez-Benítez, Pedro Jiménez-Mejías, Modesto Luceño, Santiago Martín-Bravo

Supplementary material 6 Haplotype network obtained from the statistical parsimony analysis of the combined plastid sequences of Iberian Carexsect.Phacocystis species, including *C.quixotiana*. Data type image (PDF file) Explanation note Small circles represent inferred extinct or not sampled haplotypes and lines mutational connections. https://binary.pensoft.net/file/824896This dataset is made available under the Open Database License (http://opendatacommons.org/licenses/odbl/1.0/). The Open Database License (ODbL) is a license agreement intended to allow users to freely share, modify, and use this Dataset while maintaining this same freedom for others, provided that the original source and author(s) are credited.Carmen Benítez-Benítez, Pedro Jiménez-Mejías, Modesto Luceño, Santiago Martín-Bravo

## References

[B1] Aedo C Buira A Medina L Fernández-Albert M (2017) The Iberian Vascular Flora: richness, endemicity and distribution patterns. In: Loidi J (Ed.) The Vegetation of the Iberian Peninsula. Plant and Vegetation (Vol. 12). Springer, Cham. 10.1007/978-3-319-54784-8_4

[B2] Anisimova M Gil M Dufayard JF Dessimoz C Gascuel O (2011) Survey of branch support methods demonstrates accuracy, power, and robustness of fast likelihood‐based approximation schemes. Systematic Biology 60(5): 685–699. 10.1093/sysbio/syr041 21540409 PMC3158332

[B3] Bachman S Moat J Hill AW de la Torre J Scott B (2011) Supporting Red List threat assessments with GeoCAT: Geospatial conservation assessment tool. ZooKeys 150: 117–126. 10.3897/zookeys.150.2109 PMC3234434 22207809

[B4] Benítez-Benítez C Miguez M Jiménez-Mejías P Martín-Bravo S (2017) Molecular and morphological data resurrect the long neglected *Carexlaxula* (Cyperaceae) and expand its range in the western Mediterranean. Anales del Jardin Botanico de Madrid 74(1): e057. 10.3989/ajbm.2438

[B5] Benítez‐Benítez C Escudero M Rodríguez‐Sánchez F Martín‐Bravo S Jiménez‐Mejías P (2018) Pliocene Pleistocene ecological niche evolution shapes the phylogeography of a Mediterranean plant group. Molecular Ecology 27(7): 1696–1713. 10.1111/mec.14567 29577497

[B6] Benítez-Benítez C Martín-Bravo S Bjorå CS Gebauer S Hipp AL Hoffmann MH Luceño M Pedersen TM Reznicek A Roalson E Volkova P Yano O Spalink D Jiménez-Mejías P (2021) Geographical vs. ecological diversification in CarexsectionPhacocystis (Cyperaceae): Patterns hidden behind a twisted taxonomy. Journal of Systematics and Evolution 59(4): 642–667. 10.1111/jse.12731

[B7] Berger SA Krompass D Stamatakis A (2011) Performance, accuracy, and web server for evolutionary placement of short sequence reads under maximum likelihood. Systematic Biology 60(3): 291–302. 10.1093/sysbio/syr010 21436105 PMC3078422

[B8] Buira A Cabezas F Aedo C (2020) Disentangling ecological traits related to plant endemism, rarity and conservation status in the Iberian Peninsula. Biodiversity and Conservation 29(6): 1937–1958. 10.1007/s10531-020-01957-z

[B9] Cano-Carmona E Valle-Tendero F (1996) Catalogo florístico de Sierra Quintana: Sierra morena (Andújar-Jaén). Monografías del Real Jardín Botánico de Córdoba 4: 5–73.

[B10] Chater AO (1980) *Carex* L. In: Tutin TG, Heywood VH, Burges NA, Moore DM, Walters SM, Webb DA (Eds) Flora Europaea (Vol. 5). Cambridge University Press, Cambridge, 290–323.

[B11] Clement M Posada D Crandall KA (2000) TCS: A computer program to estimate gene genealogies. Molecular Ecology 9(10): 1657–1659. 10.1046/j.1365-294x.2000.01020.x 11050560

[B12] de Cervantes M (1605) El ingenioso hidalgo Don Quijote de la Mancha. Juan de la Cuesta (Ed.), Madrid, España.

[B13] de Cervantes M (1615) Segunda parte del ingenioso hidalgo Don Quijote de la Mancha. Juan de la Cuesta (Ed.), Madrid, España.

[B14] Dean M Ashton PA (2008) Leaf surfaces as a taxonomic tool: The case of CarexsectionPhacocystis (Cyperaceae) in the British Isles. Plant Systematics and Evolution 273(1): 97–105. 10.1007/s00606-008-0029-8

[B15] Edgar RC (2004) MUSCLE: Multiple sequence alignment with high accuracy and high throughput. Nucleic Acids Research 32(5): 1792–1797. 10.1093/nar/gkh340 15034147 PMC390337

[B16] Egorova TV (1999) The sedges (*Carex* L.) of Russia and adjacent states (within the limits of the former USSR). St. Petersburg State Chemical-Pharmaceutical Academy and Missouri Botanical Garden, St. Louis.

[B17] Fernández García-Rojo C (2015) Aportación a la flora vascular en el tramo oriental y en el este del tramo central de Sierra Morena y estribaciones (Ciudad Real, Centro-Sur de la Península Ibérica). Años 1990–2015. Blancoana 24: 1–181.

[B18] Fernández García-Rojo C Salazar Mendías C (2019) Actualización del catálogo florístico de Sierra Morena oriental (centro-sur de la Península Ibérica, España). Acta Botanica Malacitana 44: 109–112. 10.24310/abm.v44i0.5399

[B19] García Río R (1999) Aportaciones a la flora de Sierra Morena (Ciudad Real, España). Botanica Complutensis 23: 115–132. https://revistas.ucm.es/index.php/BOCM/article/view/BOCM9999110115A

[B20] García Río R (2004) Flora vascular de Sierra Madrona y su entorno (Sierra Morena, Ciudad Real, España). Ecología 18: 147–214.

[B21] García Río R (2007) Flora y vegetación de interés conservacionista de Sierra Madrona y su entorno (Ciudad Real, Sierra Morena, España). Ecosistemas 16(1): 97–111.

[B22] Guindon S Dufayard JF Lefort V Anisimova M Hordijk W Gascuel O (2010) New algorithms and methods to estimate maximum likelihood phylogenies: Assessing the performance of PhyML 3.0. Systematic Biology 59(3): 307–321. 10.1093/sysbio/syq010 20525638

[B23] IUCN (2017) Guidelines for using the IUCN Red List Categories and Criteria (Vol. 13). Standards and Petitions Subcommittee. Species Survival Commission, Gland, Switzerland & Cambridge, UK, 108 pp. 10.2305/IUCN.CH.2016.RLE.3.en

[B24] Jermy AC Simpson DA Foley MJY Porter MS (2007) Sedges of the British Isles. Botanical Society of the British Isles, London.

[B25] Jiménez-Mejías P Martinetto E (2013) Toward an accurate taxonomic interpretation of *Carex* fossil fruits (Cyperaceae): A case study in section Phacocystis in the Western Palearctic. American Journal of Botany 100(8): 1580–1603. 10.3732/ajb.1200629 23926219

[B26] Jiménez Mejías P Escudero M Chaparro AJ Luceño M (2007) Novedades corológicas del género *Carex* para la Península Ibérica. Acta Botanica Malacitana 32: 305–309. 10.24310/abm.v32i0.7116

[B27] Jiménez-Mejías P Escudero M Guerra-Cárdenas S Lye KA Luceño M (2011) Taxonomic delimitation and drivers of speciation in the Ibero-North African Carexsect.Phacocystis rivershore group (*Cyperaceae*). American Journal of Botany 98(11): 1855–1867. 10.3732/ajb.1100120 22025295

[B28] Jiménez-Mejías P Hilpold A Frajman B Puşcaş M Koopman J Mesterházy A Grulich V Arnstein Lye K Martín-Bravo S (2014a) *Carexcespitosa*: Reappraisal of its distribution in Europe. Willdenowia 44(3): 327–343. 10.3372/wi.44.44303

[B29] Jiménez‐Mejías P Martín‐Bravo S Amini‐Rad M Luceño M (2014b) Disentangling the taxonomy of *Carexacuta* s.l. in the Mediterranean basin and the Middle East: Re‐evaluation of *C.panormitana* Guss. And *C.kurdica* Kük. Ex Hand.‐Mazz. Plant Biosystems 148(1): 64–73. 10.1080/11263504.2012.758675

[B30] Jiménez-Mejías P Rodríguez-Palacios GE Amini Rad M Martín-Bravo S (2015) Taxonomic notes on some problematic *Carex* (Cyperaceae) names from SW Asia. Phytotaxa 219(2): 183–189. 10.11646/phytotaxa.219.2.8

[B31] Jiménez-Mejías P Hahn M Lueders K Starr JR Brown BH Chouinard BN Chung K-S Escudero M Ford BA Ford KA Gebauer S Gehrke B Hoffmann MH Jin X-F Jung J Kim S Luceño M Maguilla E Martín-Bravo S Miguez M Molina A Naczi RFC Pender JE Reznicek AA Villaverde T Waterway MJ Wilson KL Yang J-C Zhang S Hipp AL Roalson EH (2016) Megaphylogenetic specimen-level approaches to the *Carex* (Cyperaceae) phylogeny using ITS, ETS, and matK: Implications for classification. Systematic Botany 41(3): 500–518. 10.1600/036364416X692497

[B32] Jiménez-Mejías P Calleja JA Martín-Torrijos L Otero A Martín-Bravo S (2020) Citas y apuntes corológicos de interés en ciperáceas ibéricas. Acta Botanica Malacitana 45: 231–233. 10.24310/abm.v45i.10245

[B33] Kukkonen I (1998) Cyperaceae. In: Rechinger KH (Ed.) Flora Iranica (Vol. 173). Akademische Druck- und Verlagsanstall, Graz, 307 pp.

[B34] Luceño M (1988) Notas caricológicas III. Anales del Jardin Botanico de Madrid 45(1): 189–196.

[B35] Luceño M (2008) *Carex* L. In: Castroviejo S Luceño M Galán A Mejías PJ Cabezas F Medina L (Eds) Flora Iberica (Vol.18). Plantas vasculares de la Península Ibérica e Islas Baleares. Cyperaceae. Real Jardín Botánico, (CSIC), Madrid, 109–250.

[B36] Luceño M Aedo C (1994) Taxonomic revision of the Iberian species of CarexL.sectionPhacocystis Dumort. (Cyperaceae). Botanical Journal of the Linnean Society 113: 183–214. 10.1111/j.1095-8339.1994.tb01931.x

[B37] Luceño M Jiménez-Mejías P (2008) Carexsect.Phacocystis Dumort. In: Castroviejo S Luceño M Galán A Mejías PJ Cabezas F Medina L (Eds) Flora Iberica (Vol.18). Plantas vasculares de la Península Ibérica e Islas Baleares. Cyperaceae. Real Jardín Botánico (CSIC), Madrid, 237–246.

[B38] Maguilla E Escudero M (2016) Cryptic species due to hybridization: a combined approach to describe a new species (*Carex*: Cyperaceae). PLoS ONE 12(2): e0172079. 10.1371/journal.pone.0172079 PMC5156347 27973589

[B39] Martín Blanco CJ Carrasco MA (2005) Catálogo de la flora vascular de la provincia de Ciudad Real. Monografías de la Asociación de Herbarios Ibero-Macaronésicos (Vol. 1), 581 pp.

[B40] Martín-Blanco CJ Carrasco de Salazar MA (2005) Catálogo de la flora vascular de la provincia de Ciudad Real. Monografías de la Asociación de Herbarios Ibero-Macaronésicos (Vol. 1). Asociación de Herbarios Ibero-Macaronésicos, 581 pp.

[B41] Martín-Bravo S Jiménez-Mejías P Villaverde T Escudero M Hahn M Spalink D Roalson EH Hipp AL Bruederle LP Fitzek E Ford BA Ford KA Garner M Gebauer S Hoffmann MH Jin X-F Larridon I Léveillé-Bourret É Lu Y-F Luceño M Maguilla E Márquez-Corro JI Míguez M Naczi R Reznicek AA Starr JR] (2019) A tale of worldwide success: Behind the scenes of *Carex* (Cyperaceae) biogeography and diversification. Journal of Systematics and Evolution 57(6): 695–718. 10.1111/jse.12549

[B42] Martín Herrero J Cirujano Bracamonte S Moreno Pérez M Peris Gisbert JB Stübing Martínez G (2003) La vegetación protegida en Castilla-La Mancha. Descripción, ecología y conservación de los hábitats de protección especial. Junta de Comunidades de Castilla-la Mancha, Toledo.

[B43] Miller MA Pfeiffer WT Schwartz T (2010) Creating the CIPRES Science Gateway for inference of large phylogenetic trees. Proceedings of the Gateway computing environments workshop (GCE). New Orleans, 8 pp. 10.1109/GCE.2010.5676129

[B44] Mittermeier RA Turner WR Larsen FW Brooks TM Gascon C (2011) Global Biodiversity Conservation: The Critical Role of Hotspots. In: Zachos F, Habel J (Eds) Biodiversity Hotspots. Springer, Heidelberg. 10.1007/978-3-642-20992-5_1

[B45] Muñoz Jiménez (1976) Los Montes de Toledo. Estudios de Geografía Física. Departamento de Geografía de la Universidad de Oviedo, Instituto J.S. Elcano (CSIC), Oviedo, España.

[B46] Myers N Mittermeier RA Mittermeier CG Fonseca GAB Kent J (2000) Biodiversity hotspots for conservation priorities. Nature 403(6772): 853–858. 10.1038/35002501 10706275

[B47] Nilsson Ö (1985) *Carex* L. In: Davis P (Ed.) Flora of Turkey 9.Edinburg University Press, Edinburg, 73–158.

[B48] Ramos-Gutiérrez I Lima H Pajarón S Romero-Zarco C Sáez L Pataro L Molina-Venegas R Rodríguez MA Moreno-Saiz JC (2021) Atlas of the vascular flora of the Iberian Peninsula biodiversity Hotspot (AFLIBER). Global Ecology and Biogeography 30(10): 1951–1957. 10.1111/geb.13363

[B49] Roalson EH Jiménez-Mejías P Hipp AL Benítez-Benítez C Bruederle LP Chung K-S Escudero M Ford BA Ford K Gebauer S Gehrke B Hahn M Hayat Quasim M Hoffmann MH Jin X-F Kim S Larridon I Léveillé-Bourret É Lu Y-F Luceño M Maguilla E Márquez-Corro JI Martín-Bravo S Masaki T Míguez M Naczi RFC Reznicek AA Spalink D Starr JR (2021) A framework infrageneric classification of *Carex* (Cyperaceae) and its organizing principles. Journal of Systematics and Evolution 59(4): 726–762. 10.1111/jse.12722

[B50] Sánchez-Villegas R Escudero M Martín-Bravo S Salazar-Mendías C Algarra JA Luceño M (2022) Carexcamposiisubsp.tejedensis (Cyperaceae), a new taxon for Southern Iberian Peninsula based on molecular, morphological and ecological differentiation. Mediterranean Botany (online). 10.5209/mbot.80087

[B51] Schultze-Motel W (1968) *Carex* L. In: Hegi A (Ed.) Illustrierte Flora von Mitteleuropa (3rd edn.) 1/II. P. Parey, Berlin & Hamburg, 96–274.

[B52] Stamatakis A (2014) RAxML v. 8: A tool for phylogenetic analysis and post-analysis of large phylogenies. Bioinformatics 30(9): 1312–1313. 10.1093/bioinformatics/btu033 24451623 PMC3998144

[B53] Thiers BM (2023) Index Herbariorum. https://sweetgum.nybg.org/science/ih/

[B54] Troia A Santangelo A Gianguzzi L (2018) Nomenclatural remarks on Carexsect.Sylvaticae (Cyperaceae): *C.laxula* and related names. Phytotaxa 349(1): 79–84. 10.11646/phytotaxa.349.1.10

[B55] Vargas P (2020) The Mediterranean floristic region: high diversity of plants and vegetation types. In: Goldstein MI, Dellasala DA, eds. Encyclopedia of the World’s Biomes 3. Elsevier, Amsterdam, 602–616. 10.1016/B978-0-12-409548-9.12097-4

[B56] Vicioso C (1959) Estudio monográfico sobre el género *Carex* en España. Boletín del Instituto Forestal de Investigaciones y Experiencias, 205 pp.

