# Supplementary figures and images for: ﻿Carexquixotiana (Cyperaceae), a new Iberian endemic from Don Quixote’s land (La Mancha, S Spain)
Source: PhytoKeys. 2023 Mar 14;221:161–86. doi: 10.3897/phytokeys.221.99234 (PMC10209519; doi:10.3897/phytokeys.221.99234)

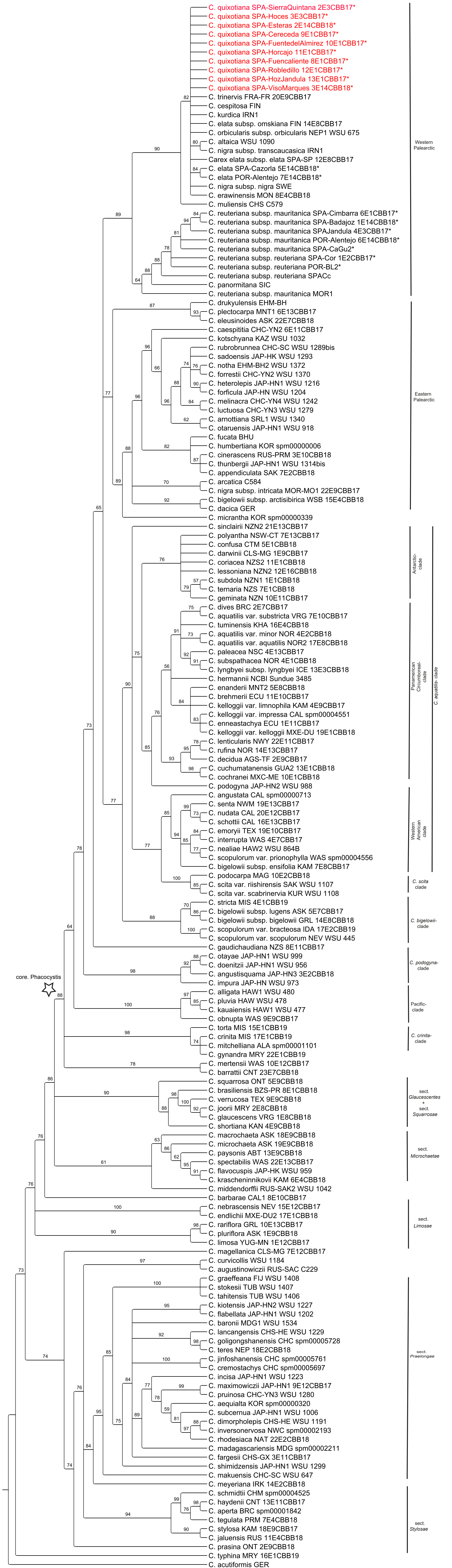

Supplement: Supplementary material 4 — Cladogram of sect. Phacocystis, including newly sampled sequences [file phytokeys-221-161_article-99234__-s004.pdf]

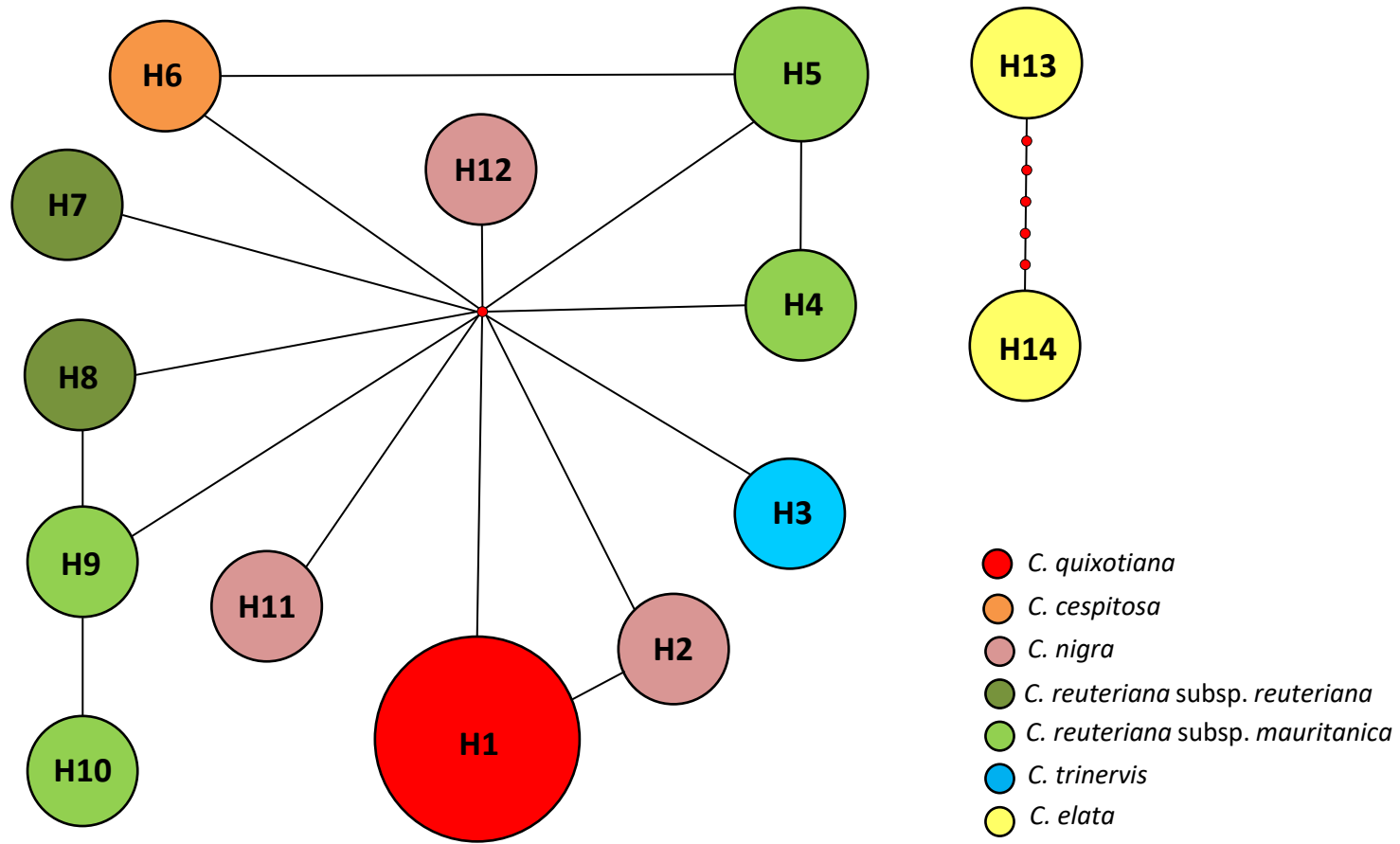

Supplement: Supplementary material 6 — Haplotype network obtained from the statistical parsimony analysis of the combined plastid sequences of Iberian Carexsect.Phacocystis species, including C.quixotiana. [file phytokeys-221-161_article-99234__-s006.pdf]
